# Donor-aware multi-cohort single-cell analysis identifies an LXR-linked tubular cholesterol-handling state in membranous nephropathy with a coordinated five-gene lipid-remodeling signature across renal tumors

**DOI:** 10.3389/fgene.2026.1916595

**Published:** 2026-10-14

**Authors:** Yalan Mao, Ran Li, Bin Li, Hongfen Pan

**Affiliations:** 1 Department of Nephrology, Tianshui the Second People’s Hospital, Tianshui Cooperation of Chinese and Western Medicine Hospital, Tianshui City, Gansu Province, China; 2 Department of Operating Room, Huai’an Hospital of Huai’an City (Huai’an Clinical Medical College of Jiangsu University), Huai’an City, Jiangsu Province, China; 3 Department of Laboratory, Tianshui the Second People’s Hospital, Tianshui Cooperation of Chinese and Western Medicine Hospital, Tianshui City, Gansu Province, China; 4 Department of Hemodialysis Center, Huzhou Wuxing Hospital of Traditional Chinese and Western Medicine, Huzhou City, Zhejiang Province, China

**Keywords:** cholesterol handling, HK-2 cells, lipid remodeling, Liver X receptor, membranous nephropathy, proteinuria, renal cancer, renal tubule

## Abstract

**Background:**

Membranous nephropathy (MN) is an immune-mediated glomerular disease in which sustained proteinuria exposes renal tubular epithelial cells to an abnormal filtered-protein load. The tubular metabolic response to this exposure remains incompletely characterized. We investigated whether MN is associated with a reproducible tubular cholesterol-handling transcriptional state and assessed its relationship to protein overload *in vitro*. We further evaluated whether an experimentally anchored five-gene subset showed a coordinated tumor-associated transcriptional shift across major renal cancer histologies.

**Methods:**

Three kidney single-cell RNA-sequencing datasets and one glomerular bulk-transcriptomic cohort were analyzed using the donor as the biological replicate. GSE241302 was used for complete-case exploratory discovery, OEP004186 for formal donor-aware support, GSE171458 for directional support, and GSE200828 for tissue-level disease context. A frozen 47-gene LXR-linked cholesterol-handling program and its nested 37-gene cholesterol transport/efflux component were evaluated by donor-level pseudobulk analysis, leave-one-donor-out and leave-one-gene-out sensitivity analyses. HK-2 cells were exposed to protein overload, followed by quantitative reverse-transcription PCR for representative transport and lipid-remodeling genes. The same experimentally anchored five-gene panel was then evaluated in 129 paired tumor–adjacent-normal samples across TCGA-KIRC, TCGA-KIRP, and TCGA-KICH to define a renal tumor-associated lipid-remodeling context.

**Results:**

Across all 3 MN donors in GSE241302, program scores were higher than the internal reference in proximal tubule (PT) and loop-of-Henle/thick ascending limb (loop/TAL) compartments. In OEP004186, the program was increased in PT (effect +0.480, false-discovery rate [FDR] 0.0128), PT-VCAM1 (+0.348, FDR 0.0165), and loop/distal epithelial cells (+0.310, FDR 0.0242), with positive effects in every leave-one-MN-donor-out analysis. GSE171458 provided concordant directional support in PT and loop/TAL. The PT-VCAM1 fraction was not increased in MN, whereas the program remained higher within both PT and PT-VCAM1, favoring a within-state transcriptional change. Sixteen program genes showed positive average effects across the three single-cell resources. In GSE200828, the overall program was higher in MN than in living donors (+0.226, FDR 0.0349) but not higher than in other glomerular diseases. Protein overload increased ABCA1, ABCG1, SREBF1, FASN, and SCD expression in HK-2 cells. Across the three renal cancer histologies, the five-gene panel showed a concordant paired increase (REML-pooled effect +0.967, 95% CI 0.843–1.092), and 85.3% of patients exhibited simultaneous increases in the cholesterol-efflux and lipid-synthesis/remodeling arms.

**Conclusion:**

MN is associated with a donor-supported LXR-linked cholesterol-handling transcriptional state concentrated in PT and loop/TAL-related epithelial compartments. The state is responsive to protein overload in HK-2 cells and may reflect a broader tubular lipid-homeostasis response to proteinuric kidney injury. The data do not establish direct LXR activation, increased cholesterol-efflux flux, or MN specificity. The paired renal cancer analysis further identifies a coordinated tumor-associated five-gene lipid-remodeling signature across KIRC, KIRP, and KICH, while not establishing tumor-cell-intrinsic LXR activation, immune effects, or a causal tumor mechanism.

## Introduction

Membranous nephropathy (MN) is an organ-specific autoimmune glomerular disease and one of the leading causes of nephrotic syndrome in adults (Ronco et al., 2021; Kidney Disease: Improving Global Outcomes KDIGO Glomerular Diseases Work Group, 2021). The identification of M-type phospholipase A2 receptor (PLA2R) and thrombospondin type-1 domain-containing 7A as target antigens transformed the diagnostic and pathogenic framework of primary MN (Beck et al., 2009; Tomas et al., 2014). More recently, tissue-based proteomics has expanded the antigenic spectrum and has reinforced the view that MN comprises biologically distinct subgroups rather than a single uniform entity (Sethi and Fervenza, 2024; Hoxha et al., 2022). Antigen status, antibody burden, proteinuria, kidney function, and the risk of spontaneous remission now inform clinical monitoring and treatment decisions (Kidney Disease: Improving Global Outcomes KDIGO Glomerular Diseases Work Group, 2021; Couser, 2017; Caravaca-Fontan et al., 2022). Even so, the mechanisms that connect glomerular immune injury with downstream parenchymal responses remain incompletely resolved.

Most mechanistic work in MN has appropriately focused on subepithelial immune deposits, complement-mediated injury, and podocyte dysfunction. Patient-level molecular profiling has also demonstrated that MN has a recognizable glomerular transcriptional signature, although substantial heterogeneity remains across individuals and disease stages (Sealfon et al., 2022). Proteinuria, however, is not simply a passive marker of glomerular barrier failure. Filtered proteins are taken up along the nephron, particularly by proximal tubular epithelial cells, and persistent overload can alter tubular signaling, redox balance, inflammatory mediator production, and cell survival (Abbate et al., 2006; Birn and Christensen, 2006; Zoja et al., 2004; Baines and Brunskill, 2011). The biological effects of urinary proteins are also influenced by their molecular cargo. Albumin-bound fatty acids can exacerbate tubular oxidative stress and tubulointerstitial injury, whereas fatty-acid-free albumin and lipid-bearing albumin may not provoke identical responses (Ruggiero et al., 2014; Thomas et al., 2002). These observations suggest that the tubular compartment may provide an important link between glomerular protein leakage and the broader metabolic consequences of nephrotic-range proteinuria.

Lipid homeostasis is markedly disturbed in nephrotic syndrome, with changes extending beyond circulating lipoprotein concentrations to lipid uptake, synthesis, transport, remodeling, and catabolism (Vaziri, 2016; Agrawal et al., 2018). Intrarenal lipid dysmetabolism and lipid-droplet accumulation have been implicated in chronic kidney injury, and tubular epithelial cells are particularly sensitive to disturbances in fatty-acid oxidation and cellular energy handling (Mitrofanova et al., 2023; Kang et al., 2015). Liver X receptors [LXRα (NR1H3) and LXRβ (NR1H2)] are sterol-responsive nuclear receptors that heterodimerize with retinoid X receptors and coordinate genes involved in cholesterol transport, efflux, and inflammatory signaling (Zelcer and Tontonoz, 2006; Calkin and Tontonoz, 2012). Among their best-characterized downstream effectors, ATP-binding cassette transporters ABCA1 and ABCG1 facilitate cellular cholesterol export and contribute to membrane cholesterol homeostasis (Ye et al., 2011). Altered expression of LXR-, SREBP-, and ABCA1-related pathways has been described in nephrotic and diabetic kidneys, and experimental LXR signaling can influence renal lipid handling and glomerular integrity (Kim and Vaziri, 2009; Tsun et al., 2014; Patel et al., 2014). Whether a comparable program is present in tubular epithelial cells in human MN has not been defined.

Single-cell RNA sequencing has begun to resolve the cellular landscape of MN kidneys and has identified immune, podocyte, endothelial, and intercellular signaling alterations (Shi et al., 2023; Cai et al., 2024; Xu et al., 2021; Gu et al., 2024). These studies have substantially broadened the field, but tubular epithelial responses have generally remained secondary to glomerular and immune-cell questions. In addition, single-cell disease comparisons are vulnerable to pseudoreplication when individual cells, rather than patients, are treated as independent biological observations (Squair et al., 2021). We therefore performed a donor-aware, multi-cohort reanalysis centered on non-podocyte compartments. Using complete-case GSE241302 for exploratory discovery, OEP004186 for formal donor-level support, GSE171458 for directional support, and GSE200828 for tissue-level context, we examined an LXR-linked cholesterol-handling program across tubular segments. We further tested whether protein overload reproduced representative components of this program in HK-2 cells. To extend this renal metabolic program into an oncologic context, we also evaluated the experimentally anchored five-gene panel in paired tumor–adjacent-normal samples from three TCGA renal cancer histologies. This cross-disease comparison was designed to determine whether the lipid-remodeling readout also marks a coordinated tumor-associated renal transcriptional state, without treating the tumor cohorts as validation of MN or as direct evidence of LXR activation.

## Methods

### Study design and analytical framework

This study integrated three kidney single-cell RNA-sequencing resources, one glomerular bulk-transcriptomic cohort, and a protein-overload experiment in human proximal tubular epithelial cells. GSE241302 was used as the complete-case exploratory discovery dataset, OEP004186 served as the principal donor-aware support cohort, GSE171458 provided directional replication, and GSE200828 defined the tissue-level disease context. The primary computational endpoint was an LXR-linked tubular cholesterol-handling transcriptional program. All formal single-cell comparisons used the donor, rather than the individual cell, as the biological replicate. Analyses of GSE241302 were treated as descriptive because the series contained only one internal normal-reference donor. No new human tissue was collected for this study. The paired TCGA kidney cancer cohorts were used solely for cross-disease renal contextualization. The renal cancer component specifically tested whether the experimentally anchored five-gene readout showed a reproducible tumor-associated shift across independent renal cancer histologies.

### Public datasets and assigned analytical roles

The datasets and experimental model, including their groups, analytical roles, and statistical units, are summarized below.

**Table:** 

Dataset	Data type	Groups	Primary analytical role	Statistical unit
GSE241302	Kidney scRNA-seq	3 MN; 1 internal normal reference	Complete-case discovery, cell-type annotation, tubular-segment analysis, and donor-direction screening	Donor; descriptive direction
OEP004186	Kidney scRNA-seq	11 MN; 7 controls	Principal donor-aware support, tubular-segment pseudobulk analysis, PT-VCAM1 abundance analysis, and robustness testing	Donor
GSE171458	Kidney scRNA-seq	6 IMN; 2 controls	Directional replication in PT, LOH/TAL, and DCT/CD	Donor
GSE200828	Glomerular microarray	51 MN; 6 living donors; 31 FSGS; 19 MCD; 46 other	Patient-level tissue context and cross-disease comparisons	Patient
HK-2 model	*In vitro* qRT-PCR	Control; protein overload	Expression-level concordance of selected cholesterol-transport and lipid-remodeling genes	Independent culture experiment

### GSE241302 acquisition, quality control, and integration

Raw gene-by-cell matrices for GSM7720822, GSM7720823, GSM7720824, and GSM7720825 were obtained from GEO accession GSE241302. All 3 MN samples and the internal normal-reference sample were included. The published filtering framework was applied separately to each donor, retaining cell-called barcodes with 200–2,500 detected genes and a mitochondrial-read proportion below 30%. Predicted doublets were identified independently in each donor using scDblFinder. Five additional extreme cross-lineage/ambient-proxy outliers were removed during a conservative contamination-sensitivity screen. Because empty-droplet matrices were unavailable, formal ambient-RNA correction with SoupX was not claimed. The final clean dataset contained 22,887 cells.

Library-size normalization was performed within donor, followed by log transformation. Highly variable genes were selected for principal-component analysis. Batch-corrected low-dimensional coordinates were used only for visualization, clustering, and annotation; unintegrated raw counts were retained for donor-level pseudobulk summaries. UMAP was used for visualization. Major cell classes were assigned using canonical kidney and immune markers, without using the target cholesterol-handling genes to define cell identities.

### Cell-type annotation and tubular segmentation in GSE241302

Major annotations included tubular epithelial, endothelial, myeloid, PEC/epithelial, lymphoid, podocyte, and mesangial/pericyte compartments. Annotation was supported by LRP2 and SLC34A1 for tubular epithelial cells; EMCN and PECAM1 for endothelial cells; LYZ and CTSS for myeloid cells; KRT19 and EPCAM for PEC/epithelial cells; CD3D and NKG7 for lymphoid cells; NPHS1 and NPHS2 for podocytes; and RGS5 and CSPG4 for mesangial/pericyte cells. Tubular epithelial cells were subsequently divided into proximal tubule (PT), loop of Henle/thick ascending limb (LOH/TAL), and collecting-duct compartments using LRP2/SLC34A1/CUBN, UMOD/SLC12A1/KCNJ1, and AQP2/SCNN1G/ATP6V1B1, respectively.

### Unbiased non-podocyte pathway screening in GSE241302

For tubular epithelial, myeloid, endothelial, and PEC/epithelial compartments, raw counts were summed separately for each donor to generate donor-by-cell-type pseudobulk profiles. Genes were ranked for each MN donor relative to the same internal reference donor using signed log-expression differences. Preranked enrichment analysis was performed against Hallmark, Reactome, and Gene Ontology biological-process collections. Because there was only one reference donor, nominal enrichment P values and false-discovery rates in this dataset were treated as exploratory and were not interpreted as patient-level disease inference. Candidate programs were prioritized according to directional concordance across all 3 MN donors, multigene contribution, biological coherence, and subsequent directional support in OEP004186.

### Definition of the LXR-linked tubular cholesterol-handling program

The primary 47-gene program was defined before the formal support analyses as the nonredundant union of two Reactome gene sets: NR1H2/NR1H3-mediated signaling and NR1H3/NR1H2-regulated gene expression linked to cholesterol transport and efflux. The second Reactome set formed a nested 37-gene transport/efflux program. Only genes measurable after dataset-specific expression filtering were used in each score. Within each dataset and tubular segment, log-normalized pseudobulk expression was standardized gene-wise across donors, and the program score was calculated as the unweighted mean of the measurable members. Representative members included ABCA1, ABCG1, APOC1, MYLIP, SREBF1, FASN, SCD, ANGPTL3, RXRA, RXRB, TNRC6A, TNRC6C, AGO1, AGO2, and TBL1X. Complete frozen membership was retained in the reproducibility files and is provided in Supplementary Table S1.

### OEP004186 donor-aware tubular analysis

The processed Seurat object, cell-level metadata, and raw RNA counts from OEP004186 were obtained from the National Genomics Data Center. The dataset contained 18 identity-resolved donors, including 11 MN and seven control donors. The deposited singlet calls and fine cell annotations were retained, and formal analysis used the raw RNA-count layer rather than the integrated expression layer. Tubular populations were harmonized as PT, PT-VCAM1, loop/distal epithelium, and collecting duct; collecting-duct principal and intercalated cells were combined for the segment-level program analysis. A donor was included for a segment when at least 20 cells of that segment were available.

Raw counts were aggregated to donor-by-segment pseudobulk profiles and normalized to log-counts per million. Program scores were calculated as described above. MN-control differences were estimated using two-sided Welch tests on donor-level scores, with 95% confidence intervals. Benjamini–Hochberg correction was applied across the prespecified tubular compartments within the dataset. Robustness was assessed by repeating the analysis after removal of each MN donor in turn. The PT-VCAM1 proportion was defined as PT-VCAM1/(PT + PT-VCAM1) within each donor and analyzed using a donor-level quasi-binomial generalized linear model; raw group means and the logit-scale disease coefficient were reported.

### GSE171458 processing and directional replication

Donor-specific raw matrices from GSE171458 were processed independently. Cells with 200–7,000 detected genes and less than 30% mitochondrial reads were retained, and scDblFinder was applied within donor. Integrated coordinates were used for visualization and annotation only. PT, LOH/TAL, and DCT/CD were analyzed using donor-by-segment pseudobulk profiles. The same frozen 47-gene program and scoring procedure were applied. Group effects and confidence intervals were calculated at the donor level. Because the dataset included only two control donors, the results were used as directional replication rather than strong independent validation.

### GSE200828 bulk glomerular analysis

The normalized glomerular expression matrix and diagnostic labels were obtained from GSE200828. All 153 samples were included: 51 MN, six living-donor references, 31 FSGS, 19 MCD, and 46 Other kidney diseases. Probe-level features were mapped to unique gene symbols, and genes represented by multiple retained features were summarized using a predefined rule. Program scores were calculated by gene-wise standardization across the full cohort followed by unweighted averaging. Diagnosis was modeled using limma, and prespecified contrasts compared MN with living donors, FSGS, MCD, Other, and the pooled non-MN disease group. Benjamini–Hochberg adjustment was applied across the defined program-by-contrast testing family. GSE200828 was interpreted as tissue-level disease context rather than as proof of tubular cellular origin.

### Renal tumor transcriptomic contextual analysis

For cross-disease renal contextualization, harmonized TCGA STAR-count expression matrices for kidney renal clear cell carcinoma (KIRC), kidney renal papillary cell carcinoma (KIRP), and kidney chromophobe (KICH) were obtained through the UCSC Xena GDC hub. Analyses were restricted to patients with matched primary-tumor and solid-tissue-normal samples (72 KIRC, 32 KIRP, and 25 KICH pairs). The experimentally anchored panel comprised ABCA1, ABCG1, SREBF1, FASN, and SCD and was fixed before the TCGA analysis. Expression for each gene was standardized within each paired cohort, and the panel score was calculated as the unweighted mean of the five standardized values. ABCA1 and ABCG1 defined the cholesterol-efflux arm, whereas SREBF1, FASN, and SCD defined the lipid-synthesis/remodeling arm. Paired tumor–normal differences were assessed using paired t-tests with Benjamini–Hochberg correction across the prespecified tests. Cohort effects were combined using restricted maximum-likelihood random-effects models. Patient-level joint states were defined from the signs of the two arm changes. Pearson correlations quantified arm coordination, and Fisher-z random-effects models summarized correlations across histologies. Principal component analysis was performed on standardized paired gene changes, and PC1 loadings were evaluated in 2,000 histology-stratified bootstrap samples. Five-dimensional joint shifts were assessed using one-sample Hotelling T2 tests. This analysis was used only for renal cancer contextualization and was not treated as an MN validation cohort. Within these prespecified boundaries, the paired analysis was used to characterize a renal tumor-associated lipid-remodeling signature and its consistency across histologies.

### Cross-dataset integration and robustness analyses

Segment-specific effects from GSE241302, OEP004186, and GSE171458 were displayed on a common directional scale. GSE241302 effects were descriptive differences between each MN donor and the single reference donor and were shown without formal confidence intervals. OEP004186 and GSE171458 effects were estimated from donor-level scores. Leave-one-gene-out analyses recalculated the program after removing each measurable member in turn; the minimum and maximum resulting effects and the proportion retaining a positive direction were reported. Cross-dataset gene-direction summaries identified members with positive average effects in all three single-cell resources. No single-gene hub was selected from these analyses.

### Inference of NR1H2/NR1H3 activity

Transcription-factor activity was inferred from donor-level pseudobulk expression using the CollecTRI regulatory network and the univariate linear-model implementation in decoupleR. NR1H2 and NR1H3 activity scores were compared between MN and control donors within OEP004186 tubular compartments. All transcription factors evaluable in the network formed the multiple-testing family. These analyses were considered exploratory because the NR1H2/NR1H3 effects did not survive correction across the complete transcription-factor space.

### HK-2 cell culture and protein-overload model

Human renal proximal tubular epithelial HK-2 cells were obtained from Procell Life Science and Technology Co., Ltd. (Wuhan, China; catalogue CL-0109). Cells were maintained in minimum essential medium containing non-essential amino acids (Procell, PM150410), supplemented with 10% fetal bovine serum (Procell, 164,210-50) and 1% penicillin-streptomycin (Procell, PB180120), at 37 °C in a humidified atmosphere containing 5% CO^2^. The identity of the cell line was authenticated by the supplier, and cultures were confirmed to be free of *mycoplasma* before use. Cells between passages three and eight after thawing were used.

For protein-overload experiments, cells were seeded in six-well plates at 2 × 10^5^ cells per well and allowed to reach approximately 70%–80% confluence. After a 12-h serum-reduction period, cells were incubated for 24 h in serum-free medium containing 20 mg/mL fatty-acid-free bovine serum albumin (Sigma-Aldrich, St. Louis, MO, United States; A7030). Control cells received the same medium without added albumin. Three independent experiments were performed using separately cultured cell preparations; each qPCR reaction was run in technical triplicate.

### RNA extraction and quantitative real-time PCR

Total RNA was isolated using TRIzol reagent (Thermo Fisher Scientific, Waltham, MA, United States; 15596026) according to the manufacturer’s instructions. RNA concentration and purity were assessed spectrophotometrically, and samples with A260/A280 values between 1.8 and 2.1 were used. One microgram of RNA was reverse-transcribed using HiScript III RT SuperMix with genomic-DNA removal (Vazyme, Nanjing, China; R323-01). Quantitative PCR was performed using ChamQ Universal SYBR qPCR Master Mix (Vazyme; Q711-02) on a QuantStudio 5 Real-Time PCR System (Thermo Fisher Scientific). Cycling conditions were 95 °C for 30 s, followed by 40 cycles of 95 °C for 10 s and 60 °C for 30 s, with a subsequent melting-curve analysis. Relative expression was calculated by the 2^−ΔΔCT^ method using GAPDH as the reference gene and the control group as calibrator. Primers were synthesized by Sangon Biotech (Shanghai, China).

### Primer sequences used for qRT-PCR

The forward and reverse primer sequences used for qRT-PCR are listed below.

**Table:** 

Gene	Forward primer (5′–3′)	Reverse primer (5′–3′)
ABCA1	TCT​CTG​CTA​TCT​CCA​ACC​TCA​TC	ACG​TCT​TCA​CCA​GGT​AAT​CTG​AA
ABCG1	CCG​ACC​GAC​GAC​ACA​GAG​A	GCA​CGA​GAC​ACC​CAC​AAA​CC
SREBF1	ACT​TCT​GGA​GGC​ATC​GCA​AGC​A	AGG​TTC​CAG​AGG​AGG​CTA​CAA​G
FASN	TTC​TAC​GGC​TCC​ACG​CTC​TTC​C	GAA​GAG​TCT​TCG​TCA​GCC​AGG​A
SCD	CCT​GGT​TTC​ACT​TGG​AGC​TGT​G	TGT​GGT​GAA​GTT​GAT​GTG​CCA​GC
GAPDH	GTC​TCC​TCT​GAC​TTC​AAC​AGC​G	ACC​ACC​CTG​TTG​CTG​TAG​CCA​A

### Statistical analysis

All formal tests were two-sided. Benjamini–Hochberg correction was used for transcriptomic multiple-testing families. Donor-level group differences in single-cell pseudobulk program scores were summarized as mean differences with 95% confidence intervals. Spearman correlation was used for nonparametric associations. GSE241302 contained only one internal normal-reference donor; therefore, its donor comparisons were reported descriptively and were not used for formal patient-level inference. For qRT-PCR, each gene was compared separately between control and protein-overload groups using a two-sided Welch t-test, followed by Holm-Šídák correction across the five genes. Data are presented as mean ± SD. Adjusted P < 0.05 was considered statistically significant. Analyses were performed in R and Python using version-controlled scripts; graphs were finalized in R and GraphPad Prism.

## Results

### Complete-case reanalysis of GSE241302 identifies a tubular cholesterol-handling candidate program

To reassess the original single-cell dataset without selectively excluding available MN samples, all 3 MN donors and the internal normal-reference donor in GSE241302 were included. Following donor-specific quality control and doublet removal, 22,887 clean cells were retained, including 1,935 cells from MN-1, 12,481 from MN-2, 5,015 from MN-3, and 3,456 from the normal-reference donor. The estimated doublet rates were 4.39%, 11.42%, 7.46%, and 5.75%, respectively, highlighting substantial donor-to-donor variation in cell recovery and data quality (Figure 1A).

**FIGURE 1:**
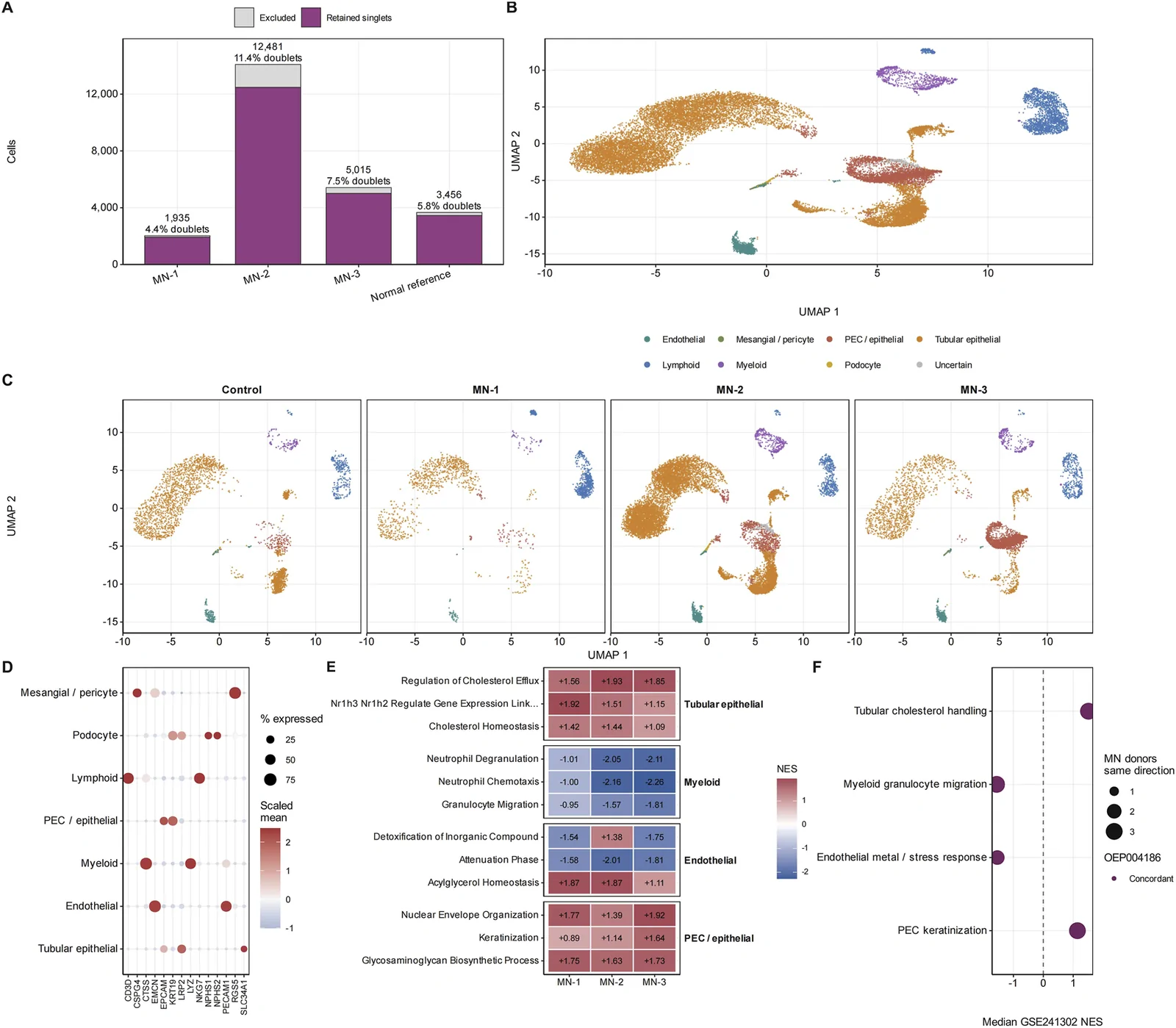
Complete-case reanalysis of GSE241302 identifies a tubular cholesterol-handling candidate program. **(A)** Donor-level quality-control summary showing retained clean cells, excluded cells, and doublet rates for the 3 MN donors and the internal normal-reference donor. **(B)** UMAP of 22,887 clean cells colored by major cell type. **(C)** Split UMAP showing the contribution of each donor in the shared embedding. **(D)** Dot plot of canonical markers used to annotate the major kidney and immune-cell compartments. Dot size represents the fraction of cells expressing the gene, and color represents scaled mean expression. **(E)** Heatmap of donor-specific normalized enrichment scores for selected pathways identified through unbiased analysis of tubular epithelial, myeloid, endothelial, and PEC/epithelial compartments. Each MN donor was compared separately with the same internal reference donor. **(F)** Ranking of four candidate cell-state programs according to their median effect in GSE241302, the number of MN donors showing the same direction, and the corresponding direction in OEP004186. Because GSE241302 contained only one internal reference donor, **(E,F)** are descriptive and do not represent formal patient-level disease inference.

The clean-cell atlas comprised 15,190 tubular epithelial cells, 3,300 PEC/epithelial cells, 2,101 lymphoid cells, 1,090 endothelial cells, and 849 myeloid cells. By contrast, only 66 podocytes and 69 mesangial/pericyte cells were retained, with most podocytes originating from a single MN donor. UMAP visualization and canonical marker expression supported the major cell-type assignments, including LRP2 and SLC34A1 in tubular epithelial cells, EMCN and PECAM1 in endothelial cells, LYZ and CTSS in myeloid cells, KRT19 and EPCAM in PEC/epithelial cells, and CD3D and NKG7 in lymphoid populations (Figures 1B–D). These findings supported a shift from the original podocyte-centred analysis toward the more extensively represented non-podocyte compartments.

Unbiased pathway analysis was subsequently performed separately for tubular epithelial, myeloid, endothelial, and PEC/epithelial compartments, with each MN donor compared independently with the internal reference donor. In tubular epithelial cells, cholesterol-related programs consistently showed positive enrichment across all 3 MN donors. The regulation of cholesterol efflux program yielded normalized enrichment scores (NESs) of 1.560, 1.935, and 1.854 in MN-1, MN-2, and MN-3, respectively. Similarly, the NR1H3/NR1H2-regulated cholesterol transport and efflux program was positively enriched in all three donors, with NESs of 1.921, 1.511, and 1.150. Hallmark cholesterol homeostasis was also directionally positive in all three donors, although the magnitude of enrichment varied between individuals (Figure 1E).

Alternative non-podocyte candidate programs showed less consistent cross-dataset potential. Myeloid granulocyte migration, neutrophil chemotaxis, and neutrophil degranulation were negatively enriched across the 3 MN donors, whereas endothelial metal- and stress-related responses showed donor-dependent directionality. PEC/epithelial keratinization and glycosaminoglycan-related programs were generally positive but showed weaker corrected statistical support. Integrating donor-level directional consistency with the subsequent OEP004186 analysis therefore prioritized the tubular lipid/cholesterol-handling program as the leading candidate for further evaluation (Figure 1F).

### The LXR-linked cholesterol-handling state spans proximal tubular and loop/TAL-related compartments in GSE241302

Tubular epithelial cells were further resolved into PT cells (9,228 cells), loop-of-Henle/TAL cells (3,439 cells), and collecting-duct cells (1,903 cells). Their identities were supported by segment-specific markers, including LRP2, SLC34A1, and CUBN in PT; UMOD, SLC12A1, and KCNJ1 in loop/TAL cells; and AQP2, SCNN1G, and ATP6V1B1 in collecting-duct populations (Figures 2A,B).

**FIGURE 2:**
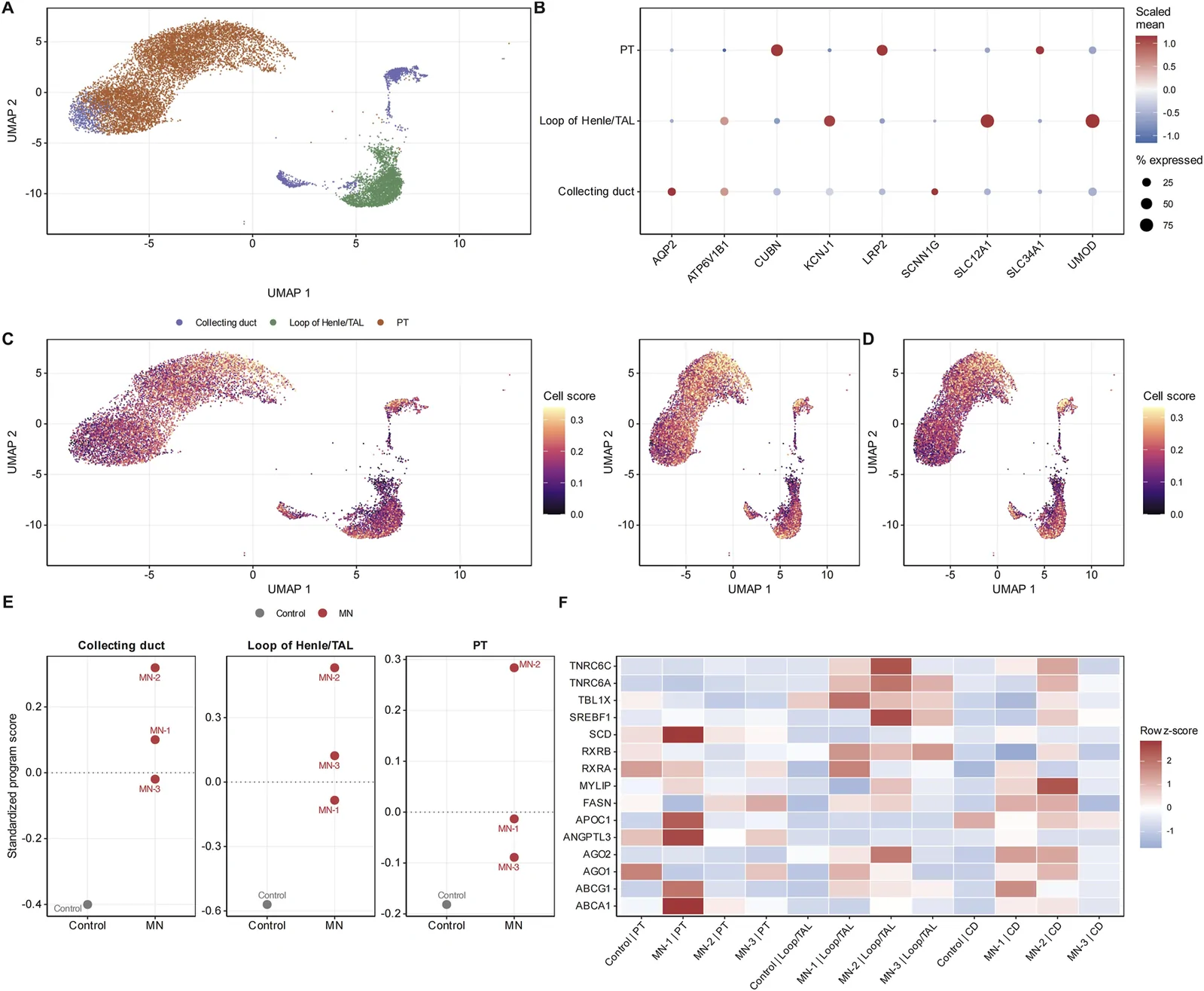
Segment-resolved distribution and molecular architecture of the LXR-linked cholesterol-handling program in GSE241302. **(A)** UMAP of tubular epithelial cells showing proximal tubule, loop-of-Henle/TAL, and collecting-duct compartments. **(B)** Dot plot of canonical tubular-segment markers. **(C)** Cellular distribution of the 47-gene LXR-linked cholesterol-handling score in the tubular UMAP. **(D)** Side-by-side feature plots of the overall 47-gene program and the nested 37-gene cholesterol transport/efflux program, displayed using the same color scale. **(E)** Donor-level program scores in PT, loop/TAL, and collecting-duct compartments. Each point represents one donor. The 3 MN donors are shown relative to the single internal normal-reference donor; no formal disease P values were calculated. **(F)** Row-scaled pseudobulk expression heatmap of representative cholesterol transport/efflux, lipid synthesis/remodeling, and LXR/RXR-associated regulatory genes across donors and tubular segments.

The 47-gene LXR-linked cholesterol-handling score was detectable across the major tubular compartments and showed a continuous distribution rather than confinement to a small discrete cell cluster (Figure 2C). A nested 37-gene cholesterol transport/efflux score showed a closely overlapping spatial pattern, indicating that cholesterol transport and efflux constituted a major component of the broader program while not representing an independent cellular state (Figure 2D).

At the donor level, all 3 MN donors showed higher program scores than the internal reference donor in each evaluated tubular compartment. In PT cells, the MN-minus-reference differences were +0.168, +0.465, and +0.093. In loop/TAL cells, the corresponding differences were +0.485, +1.102, and +0.693, whereas collecting-duct differences were +0.501, +0.719, and +0.381 (Figure 2E). The largest descriptive shift was therefore observed in the loop/TAL compartment. Because only one internal reference donor was available, these results were interpreted as donor-consistent directional observations rather than formal patient-level disease comparisons.

Expression heatmaps further showed coordinated but heterogeneous contributions from cholesterol transport genes, including ABCA1, ABCG1, APOC1, and MYLIP; lipid synthesis and remodeling genes, including SREBF1, FASN, SCD, and ANGPTL3; and LXR/RXR-associated regulatory components, including RXRA, RXRB, TNRC6A, TNRC6C, AGO1, AGO2, and TBL1X (Figure 2F). No single gene was uniformly dominant across all donors and tubular segments, supporting a distributed multigene state rather than a single-gene axis.

### Donor-aware analysis in OEP004186 supports the tubular cholesterol-handling state

The tubular program was formally evaluated in OEP004186, which included clearly annotated independent donors and substantially greater tubular cell coverage. The analysis included 41,732 PT cells, 3,141 PT-VCAM1 cells, 3,970 loop/distal cells, and 4,193 collecting-duct cells from donors who met the prespecified cell-count criteria. Canonical marker expression confirmed the PT, PT-VCAM1, loop/distal, collecting-duct principal-cell, and intercalated-cell annotations (Figures 3A,B).

**FIGURE 3:**
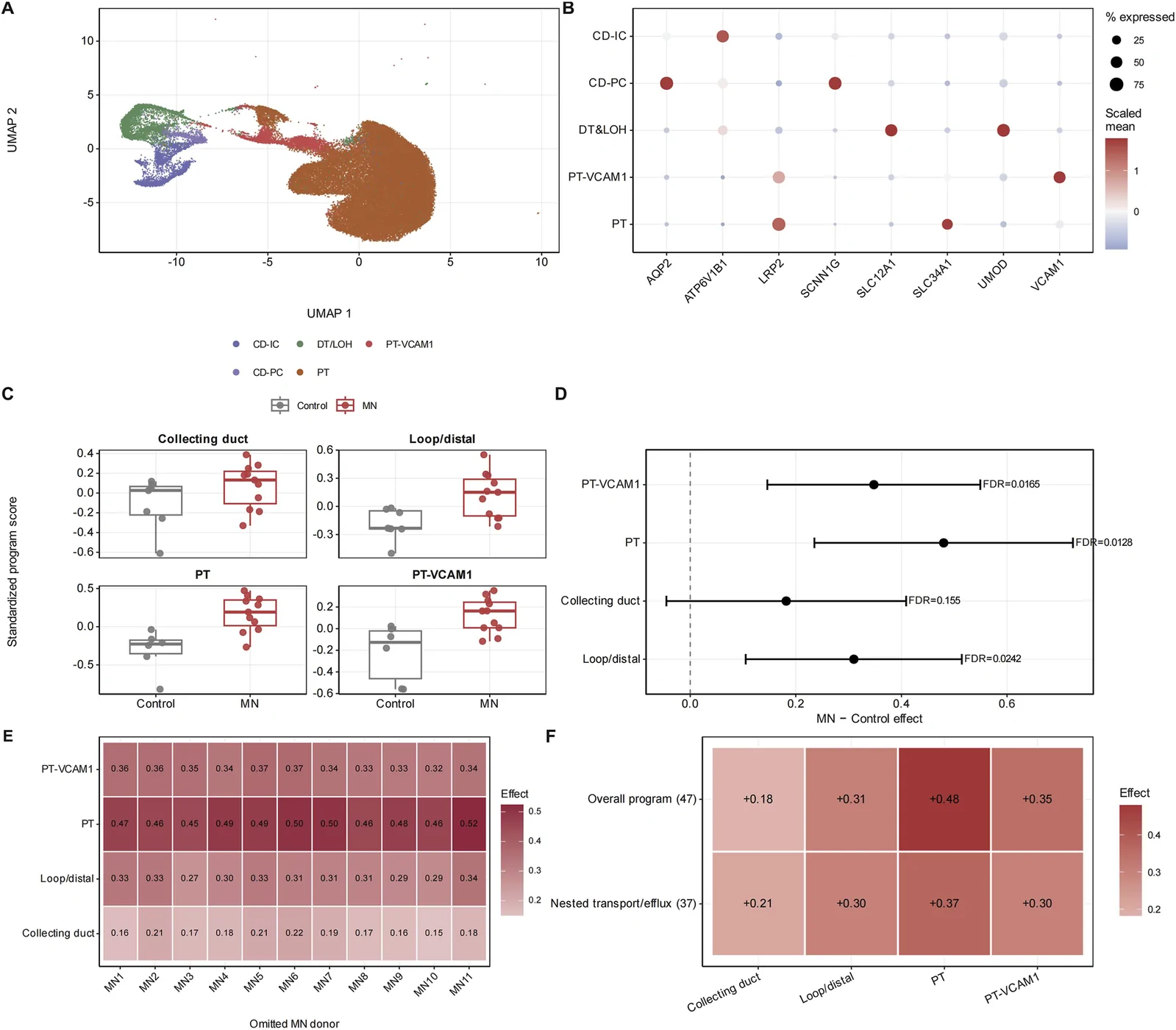
Donor-aware validation of the tubular cholesterol-handling state in OEP004186. **(A)** UMAP of OEP004186 tubular epithelial cells showing PT, PT-VCAM1, loop/distal, collecting-duct principal-cell, and intercalated-cell populations. **(B)** Dot plot of segment- and state-specific marker genes. **(C)** Donor-level cholesterol-handling program scores in PT, PT-VCAM1, loop/distal, and collecting-duct cells. Each point represents one independent donor. **(D)** Forest plot showing the MN-versus-control program effect, 95% confidence interval, nominal P value, and within-dataset Benjamini–Hochberg-adjusted FDR for each tubular compartment. **(E)** Leave-one-MN-donor-out effects. Each column represents an analysis performed after excluding 1 MN donor. **(F)** Effects of the overall 47-gene program and the nested 37-gene cholesterol transport/efflux program across tubular compartments. Positive values indicate higher donor-level scores in MN.

Donor-level pseudobulk scores were significantly higher in MN than in controls in PT, PT-VCAM1, and loop/distal epithelial cells. In PT, the mean score increased from −0.311 in controls to +0.169 in MN, corresponding to an effect of +0.480 (95% CI, +0.235 to +0.725; P = 0.00160; FDR = 0.0128). In PT-VCAM1 cells, the effect was +0.348 (95% CI, +0.146 to +0.549; P = 0.00412; FDR = 0.0165). Loop/distal cells showed an effect of +0.310 (95% CI, +0.105 to +0.514; P = 0.00907; FDR = 0.0242). Collecting-duct cells showed a smaller positive estimate of +0.182, but the confidence interval crossed zero (95% CI, −0.045 to +0.409; P = 0.136; FDR = 0.155) (Figures 3C,D).

Leave-one-MN-donor-out analysis demonstrated that the positive direction was not attributable to a single MN donor. The effects remained positive in all 11 iterations for PT (+0.450 to +0.524), PT-VCAM1 (+0.325 to +0.372), loop/distal cells (+0.267 to +0.343), and collecting-duct cells (+0.150 to +0.222) (Figure 3E). Although the collecting-duct direction was stable, its original donor-level comparison remained nonsignificant and was therefore not considered part of the reproducible core finding.

The overall 47-gene program and the nested cholesterol transport/efflux program were concordantly elevated in the three supported tubular compartments. In PT, the overall and nested-program effects were +0.480 and +0.371, respectively. Corresponding effects were +0.348 and +0.302 in PT-VCAM1 cells and +0.310 and +0.301 in loop/distal cells. Both programs passed within-dataset multiple-testing correction in these compartments, whereas neither reached significance in collecting-duct cells (Figure 3F). These findings indicated that the donor-level signal was not produced solely by genes outside the cholesterol transport/efflux component.

### Cross-dataset support is concentrated in PT and loop/TAL compartments and is not explained by PT-VCAM1 expansion

The tubular state was next assessed in GSE171458, which contained 6 IMN and 2 control donors. PT, LOH/TAL, and DCT/CD contained 7,334, 2,016, and 2,024 cells, respectively, and formed distinguishable transcriptional compartments (Figure 4A). Donor-level program effects were positive in PT (+0.215) and LOH/TAL (+0.172), but confidence intervals were wide because only two control donors were available. DCT/CD showed a small negative effect of −0.041, providing no directional support for a uniformly elevated distal/collecting-duct state (Figure 4B).

**FIGURE 4:**
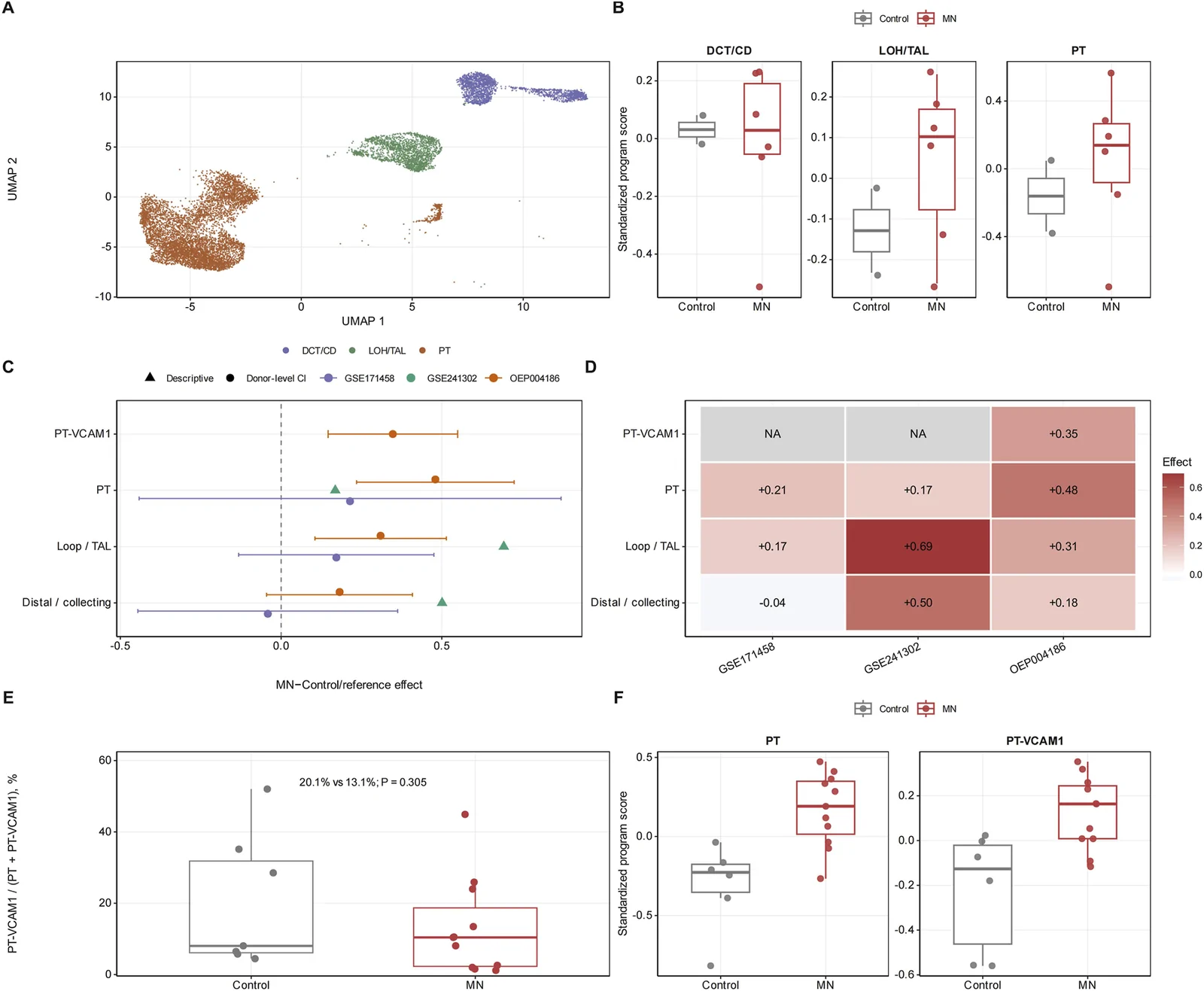
Cross-dataset support for PT and loop/TAL localization and independence from PT-VCAM1 expansion. **(A)** UMAP of the GSE171458 tubular compartment showing PT, LOH/TAL, and DCT/CD cells. **(B)** Donor-level cholesterol-handling program scores in GSE171458. Each point represents one donor; effects were calculated using donor-level pseudobulk data. **(C)** Cross-dataset forest plot of segment-specific effects in GSE241302, OEP004186, and GSE171458. GSE241302 estimates are descriptive and are shown without formal confidence intervals. **(D)** Dataset-by-segment heatmap of the observed program effects. Missing combinations reflect the absence of a directly corresponding annotation. **(E)** Donor-level PT-VCAM1 fraction, calculated as PT-VCAM1/(PT + PT-VCAM1), in control and MN samples from OEP004186. **(F)** Donor-level program scores within conventional PT and PT-VCAM1 cells. The positive effect in both populations, together with the absence of PT-VCAM1 expansion, supports an intra-subtype transcriptional-state difference rather than a change driven by PT-VCAM1 abundance.

Cross-dataset comparison demonstrated consistent positive estimates in PT across GSE241302 (+0.168), OEP004186 (+0.480), and GSE171458 (+0.215). Loop/TAL-related compartments were also positive in all three datasets, with effects of +0.693, +0.310, and +0.172, respectively. In contrast, distal/collecting-duct estimates were +0.501 in GSE241302, +0.182 in OEP004186, and −0.041 in GSE171458 (Figures 4C,D). The most reproducible anatomical range of the program was therefore restricted to PT and loop/TAL-related epithelial compartments.

To determine whether the increased score merely reflected expansion of the PT-VCAM1 injury-associated state, the proportion of PT-VCAM1 cells among all PT and PT-VCAM1 cells was evaluated at the donor level. The mean proportion was 20.06% in controls and 13.14% in MN, corresponding to an unadjusted difference of −6.91 percentage points and a logit-scale effect of −0.688 (P = 0.305). The effect remained negative in every leave-one-MN-donor-out analysis (Figure 4E). Thus, no evidence of PT-VCAM1 expansion was observed.

In contrast, the cholesterol-handling program was significantly increased within both conventional PT cells and PT-VCAM1 cells. The within-subtype effects were +0.480 in PT (P = 0.00160; FDR = 0.0128) and +0.348 in PT-VCAM1 cells (P = 0.00412; FDR = 0.0165) (Figure 4F). Together, these findings supported an intra-subtype transcriptional-state change rather than a compositional increase in PT-VCAM1 cells.

### The tubular state is supported by a distributed multigene architecture and occurs in a broader proteinuric-disease context

Gene-level effects supported coordinated contributions from cholesterol transport/efflux, lipid synthesis/remodeling, and LXR-associated regulatory components. In OEP PT cells, 29 of 38 measurable program members were positively shifted, including five of seven transport genes, four of five synthesis/remodeling genes, and 20 of 26 LXR-associated regulatory genes. Similar positive proportions were observed in PT-VCAM1 and loop/distal compartments. GSE241302 PT and loop/TAL compartments also showed predominantly positive multigene patterns, whereas GSE171458 showed greater gene-level heterogeneity despite maintaining a positive aggregate score in PT and LOH/TAL (Figure 5A).

**FIGURE 5:**
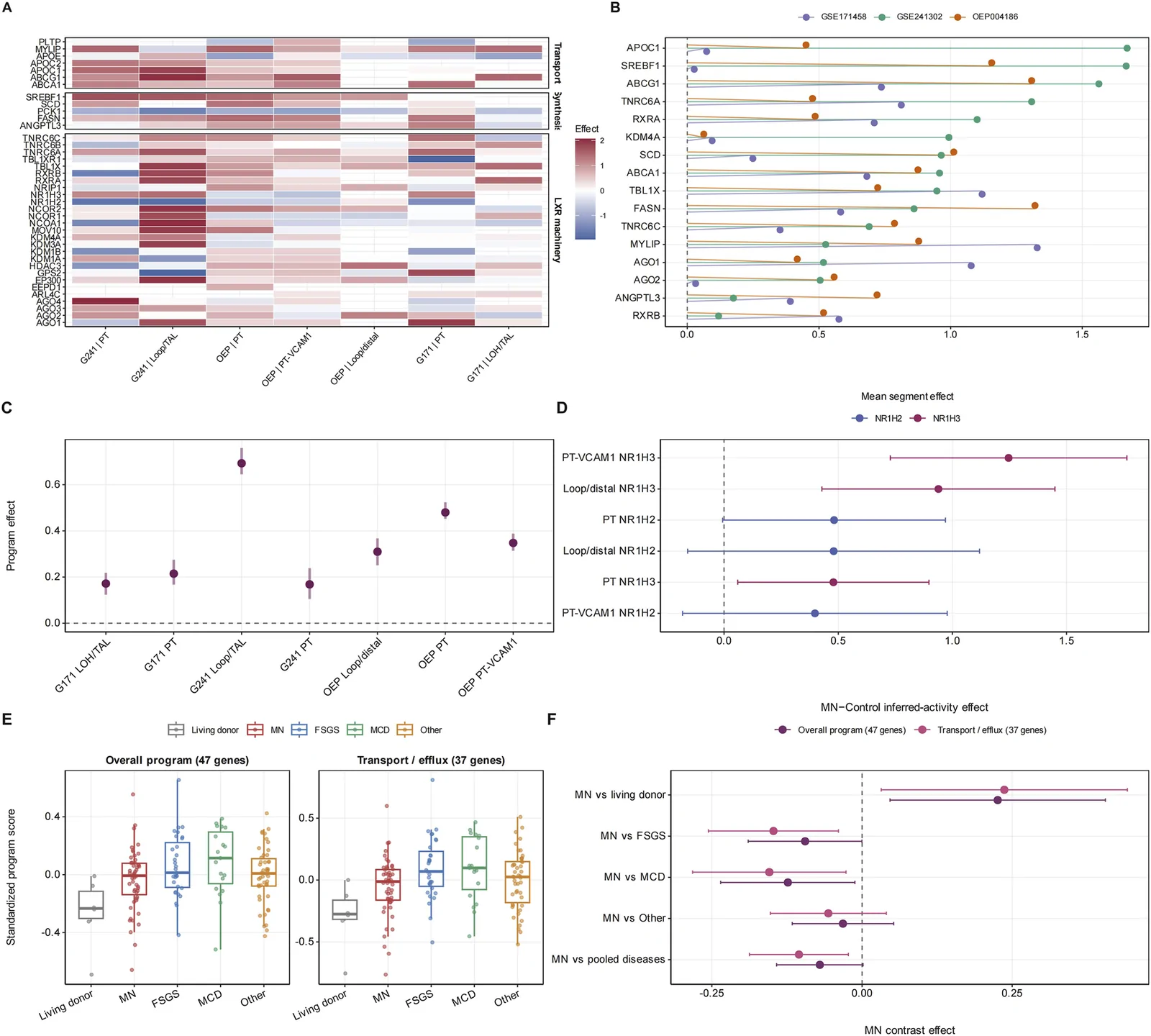
Multigene architecture, inferred LXR linkage, and tissue-level disease context. **(A)** Heatmap of the effects of the 47 program genes across the principal dataset-by-segment combinations. Genes are grouped according to cholesterol transport/efflux, lipid synthesis/remodeling, or LXR-associated regulatory function. **(B)** Effects of the 16 program genes with positive average directions across GSE241302, OEP004186, and GSE171458. **(C)** Leave-one-gene-out sensitivity analysis. Points indicate the full-program effect and ranges indicate the minimum and maximum effects after removal of each measurable program gene in turn. **(D)** Exploratory inferred NR1H2 and NR1H3 activity effects in OEP004186 tubular compartments. These estimates did not survive correction across the complete transcription-factor testing space and are presented as supportive evidence for an LXR-linked, rather than experimentally confirmed LXR-activated, state. **(E)** Overall cholesterol-handling program scores across living-donor, MN, FSGS, MCD, and Other samples in GSE200828. Each point represents one patient. **(F)** Forest plot of the overall program and nested transport/efflux program in 5 MN comparisons. Positive effects in MN versus living donors, but not versus other glomerular diseases, indicate an MN-associated rather than MN-specific tissue context.

Sixteen genes had positive average effects across all three single-cell datasets: APOC1, SREBF1, ABCG1, TNRC6A, RXRA, KDM4A, SCD, ABCA1, TBL1X, FASN, TNRC6C, MYLIP, AGO1, AGO2, ANGPTL3, and RXRB (Figure 5B). These genes represented multiple functional components of the program rather than a single dominant pathway node. For example, ABCA1 and ABCG1 represented cholesterol transport/efflux, SREBF1, FASN, and SCD represented lipid synthesis and remodeling, and RXRA/RXRB contributed to the LXR/RXR-associated transcriptional architecture.

The aggregate program remained positive after removal of any individual measurable gene. The leave-one-gene-out effect ranges were +0.105 to +0.238 in GSE241302 PT, +0.645 to +0.759 in GSE241302 loop/TAL, +0.452 to +0.524 in OEP PT, +0.314 to +0.388 in OEP PT-VCAM1, and +0.251 to +0.367 in OEP loop/distal cells. Positive directions were also retained in GSE171458 PT and LOH/TAL, although the limited donor number prevented significant replication in that dataset (Figure 5C).

Inferred NR1H3 activity was positively shifted in OEP PT (+0.479), PT-VCAM1 (+1.246), and loop/distal cells (+0.939), with nominal significance in all three compartments. NR1H2 activity was also directionally positive, but the estimates were weaker. None of the NR1H2/NR1H3 activity effects survived correction across the complete transcription-factor testing space. Accordingly, these results supported the term “LXR-linked transcriptional state” but did not establish functional LXR activation or an NR1H3-driven regulatory mechanism (Figure 5D).

At the tissue level, the overall program score in GSE200828 was higher in MN than in living donors. The MN-versus-living-donor effect was +0.226 (95% CI, +0.047 to +0.405; P = 0.0147; FDR = 0.0349). However, MN did not show higher scores than FSGS, MCD, Other, or the pooled non-MN disease group. The nested transport/efflux program showed a similar pattern, with a positive MN-versus-living-donor effect of +0.237 but no evidence of relative enrichment over the other glomerular disease groups (Figures 5E,F). These data placed the tubular state within the broader context of proteinuric or diseased kidneys rather than identifying an MN-specific signature.

### Protein overload induces cholesterol transport and lipid-remodeling genes in HK-2 cells

To determine whether a protein-overload condition could induce transcriptional changes concordant with the computationally defined tubular state, the expression of five representative program genes was measured in HK-2 cells. Protein overload increased ABCA1 expression from 1.02 ± 0.07 to 1.68 ± 0.10, corresponding to a 1.66-fold increase (Holm–Šídák-adjusted P = 0.0058). ABCG1 increased from 1.01 ± 0.05 to 1.43 ± 0.09 (1.42-fold; adjusted P = 0.0170).

The lipid synthesis and remodeling genes showed concordant changes. SREBF1 increased from 1.00 ± 0.08 to 1.33 ± 0.09 (1.33-fold; adjusted P = 0.0170), FASN increased from 1.00 ± 0.06 to 1.72 ± 0.11 (1.71-fold; adjusted P = 0.0093), and SCD increased from 1.01 ± 0.07 to 1.38 ± 0.09 (1.37-fold; adjusted P = 0.0170) (Figure 6A). Thus, protein overload coordinately induced genes representing both the cholesterol transport/efflux and lipid synthesis/remodeling components of the identified program.

**FIGURE 6:**
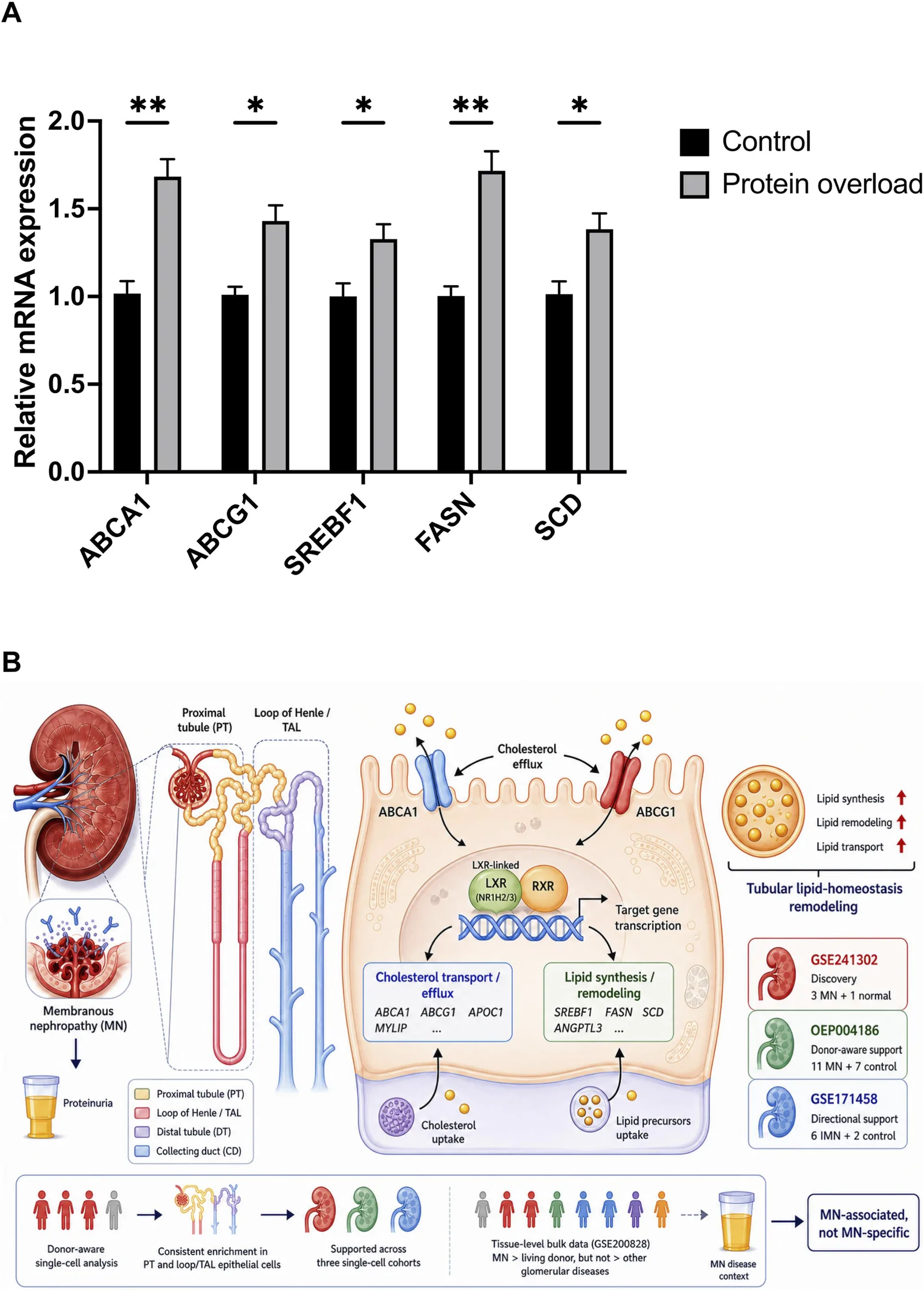
Protein-overload-induced expression of cholesterol-handling genes in HK-2 cells and integrated working model. **(A)** Relative mRNA expression of ABCA1, ABCG1, SREBF1, FASN, and SCD in control and protein-overload-treated HK-2 cells. Grouped bars represent mean ± SD from three independent culture experiments per group. Statistical comparisons were performed separately for each gene using two-sided Welch t-tests followed by Holm–Šídák correction for five comparisons. *Adjusted P < 0.05; **adjusted P < 0.01. ABCA1 and FASN met the **P < 0.01 threshold; ABCG1, SREBF1, and SCD met the *P < 0.05 threshold. **(B)** Medical illustration summarizing the proposed MN-associated tubular LXR-linked cholesterol-handling state. The schematic emphasizes PT and loop/TAL-related epithelial compartments and depicts representative cholesterol transport/efflux genes (ABCA1, ABCG1, APOC1, and MYLIP) and lipid synthesis/remodeling genes (SREBF1, FASN, SCD, and ANGPTL3). The illustration summarizes integrated transcriptional and qPCR findings and does not demonstrate direct LXR protein activation, increased cholesterol-efflux flux, or causality in MN progression.

Together with the donor-aware single-cell results, these HK-2 data provided experimental concordance for a protein-overload-responsive tubular cholesterol-handling transcriptional state. The integrated working model is summarized in Figure 6B. This model depicts coordinated cholesterol transport/efflux and lipid remodeling in PT and loop/TAL-related epithelial compartments; it should not be interpreted as direct evidence of LXR protein activation, increased cholesterol-efflux flux, or a causal effect on MN progression. The biological relationships represented in the schematic are based on the integrated computational and expression findings.

### Patient-resolved renal tumor lipid-remodeling analysis

To determine whether the experimentally anchored five-gene panel reflected a broader renal pathological response, we analyzed paired primary-tumor and adjacent-normal samples from TCGA-KIRC (n = 72), TCGA-KIRP (n = 32), and TCGA-KICH (n = 25). The five-gene panel was increased in all three histologies, with standardized paired effects of +0.916, +0.912, and +1.129, respectively, and a REML-pooled effect of +0.967 (95% CI, 0.843–1.092; P = 2.02 × 10−52). ABCA1 and ABCG1 were summarized as a cholesterol-efflux arm, whereas SREBF1, FASN, and SCD formed a lipid-synthesis/remodeling arm. Both arms increased in every histology and were positively correlated at the patient level (r = 0.509; P = 7.07 × 10−10); 110 of 129 paired patients (85.3%) showed concordant increases in both arms. A common latent factor explained 45.2% of the five-gene variance and retained uniformly positive loadings across 2,000 histology-stratified bootstrap samples. Joint five-dimensional shifts were significant in KIRC, KIRP, and KICH after Benjamini–Hochberg correction. These findings place the qPCR-anchored panel within a broader renal lipid-remodeling context, without constituting independent validation of MN or direct evidence of LXR activation (Figure 7). Accordingly, the paired tumor–normal data identify a reproducible five-gene renal tumor-associated lipid-remodeling signature shared directionally across the three histologies.

**FIGURE 7:**
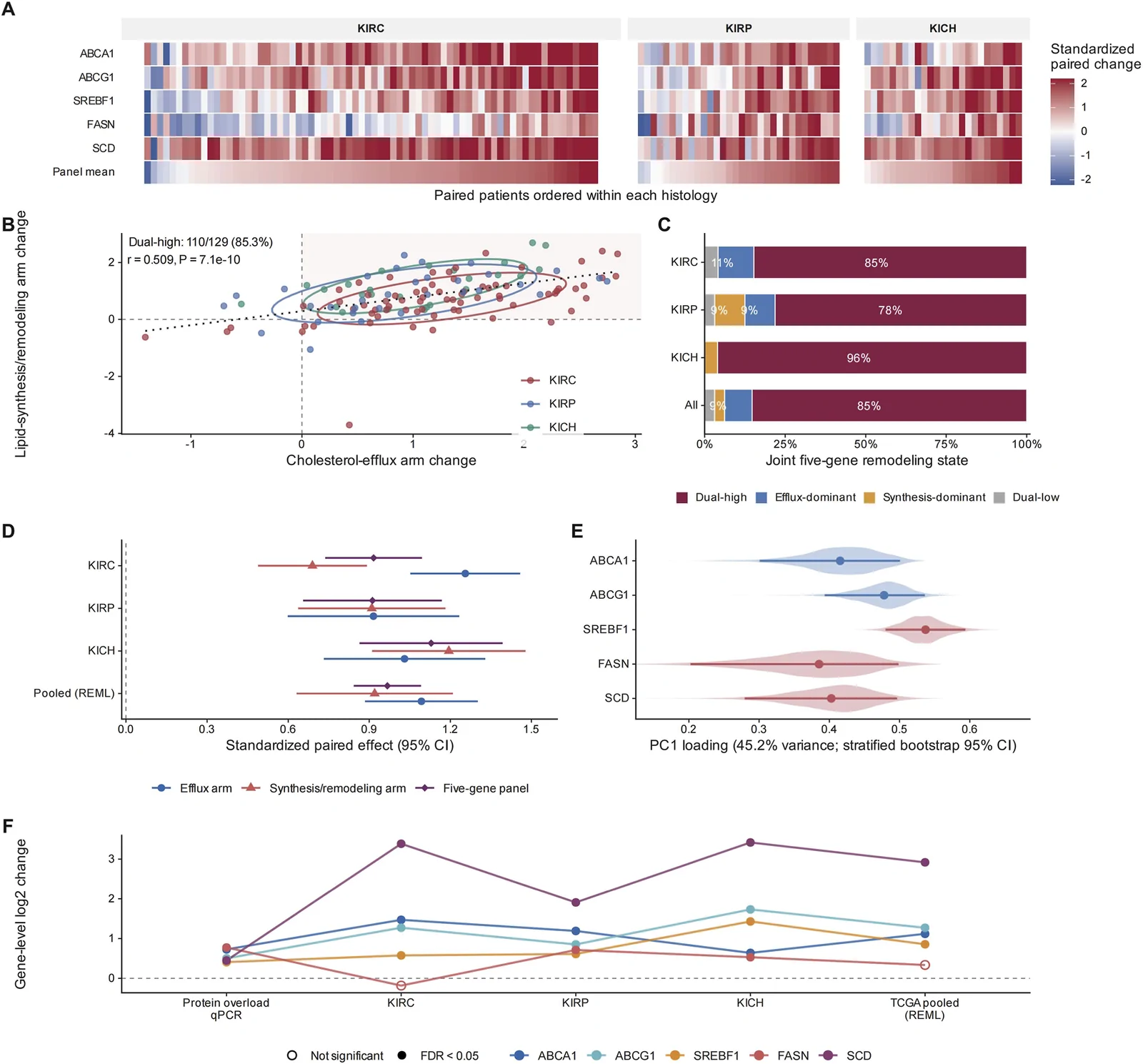
Patient-resolved analysis identifies a coordinated dual-arm renal lipid-remodeling state across three renal cancer histologies. **(A)** Heatmap of standardized paired tumor–normal changes in ABCA1, ABCG1, SREBF1, FASN, SCD, and the five-gene panel mean across 129 paired patients. Patients were ordered by the panel mean within each histology. **(B)** Patient-level relationship between the cholesterol-efflux arm (ABCA1 and ABCG1) and the lipid-synthesis/remodeling arm (SREBF1, FASN, and SCD). Ellipses enclose 68% of the fitted distribution for each histology. **(C)** Distribution of concordant dual-high, efflux-dominant, synthesis-dominant, and dual-low states. **(D)** Cohort-specific and REML-pooled effects for both functional arms and the complete five-gene panel. **(E)** Stratified-bootstrap distributions of PC1 loadings; points and horizontal intervals denote observed loadings and bootstrap 95% confidence intervals. **(F)** Gene-level effect trajectories across the protein-overload qPCR experiment, paired TCGA cohorts, and gene-specific random-effects estimates. Filled symbols indicate Benjamini–Hochberg-adjusted significance. These findings describe a coordinated cross-disease renal lipid-remodeling context and do not establish LXR activation or validate a tumor mechanism in membranous nephropathy. In an oncologic context, this figure supports a reproducible renal tumor-associated lipid-remodeling signature while preserving the stated mechanistic limitations.

## Discussion

This study identifies a reproducible tubular cholesterol-handling transcriptional state associated with MN. The central observation was not confined to one dataset or one tubular subtype. In the complete GSE241302 cohort, all 3 MN donors showed positive shifts in PT and loop/TAL-related compartments relative to the internal reference donor. OEP004186 then provided formal donor-level support in PT, PT-VCAM1, and loop/distal epithelial cells, with stable positive effects after removal of each MN donor in turn. GSE171458 showed the same direction in PT and loop/TAL, although its small control group limited precision. The signal was distributed across genes involved in cholesterol transport, efflux, lipid synthesis, and lipid remodeling, and it remained positive after individual program genes were removed. Protein overload also increased ABCA1, ABCG1, SREBF1, FASN, and SCD expression in HK-2 cells. Taken together, these findings define a protein-overload-responsive tubular lipid-homeostasis state rather than a single-gene pathway.

The tubular location of this signal is biologically plausible in a disease dominated clinically by proteinuria. Excess filtered proteins are reabsorbed and processed by tubular epithelial cells, and persistent protein exposure can initiate inflammatory, oxidative, and profibrotic responses that contribute to tubulointerstitial injury (Abbate et al., 2006; Birn and Christensen, 2006; Zoja et al., 2004; Baines and Brunskill, 2011). Albumin also carries fatty acids and other hydrophobic molecules, which can modify its cellular effects. Experimental work has shown that lipid-bearing albumin can produce greater oxidative and apoptotic stress than fatty-acid-free albumin and can aggravate protein-overload injury *in vivo* (Ruggiero et al., 2014; Thomas et al., 2002). Our data do not imply that the tubular program is necessarily injurious. The simultaneous increase in transport/efflux genes and lipid-synthesis/remodeling genes could reflect compensation, adaptation, or an early maladaptive transition. Nevertheless, the convergence of patient-derived single-cell data and the HK-2 protein-overload model supports a direct relationship between filtered-protein stress and tubular lipid-regulatory transcription.

The term “LXR-linked” was deliberately chosen to describe the architecture of the program without equating transcript abundance with receptor activation. LXRs function as sterol sensors and coordinate cholesterol handling through RXR-dependent transcriptional complexes (Zelcer and Tontonoz, 2006; Calkin and Tontonoz, 2012). ABCA1 and ABCG1 are established mediators of cholesterol export, whereas SREBF1, FASN, and SCD participate in lipid synthesis and membrane-lipid remodeling (Ye et al., 2011). In our analysis, these two functional sides moved together: ABCA1 and ABCG1 increased, but so did SREBF1, FASN, and SCD. This pattern is more consistent with coordinated lipid-homeostasis remodeling than with a simple increase in net cholesterol efflux. Similar combinations of altered LXR/SREBP signaling and cholesterol-transport machinery have been described in nephrotic and diabetic kidneys (Kim and Vaziri, 2009; Tsun et al., 2014). Experimental LXR modulation can protect renal structure in diabetic models (Patel et al., 2014), but those findings cannot be directly transferred to MN or to tubular epithelium. Direct assessment of LXR nuclear occupancy, target-gene binding, transporter protein abundance, and cholesterol flux would be required before functional activation could be claimed.

The anatomical consistency of the state was strongest in PT and loop/TAL-related compartments. PT cells are exposed to the highest filtered-protein load and have substantial energetic and membrane-transport demands. Their dependence on mitochondrial metabolism and fatty-acid oxidation may make them particularly responsive to altered lipid availability (Birn and Christensen, 2006; Baines and Brunskill, 2011; Kang et al., 2015). The loop/TAL finding was less expected but was directionally consistent across the three single-cell resources. TAL cells also have high transport activity and energy requirements, and the observed program may represent a broader epithelial response to nephrotic or proteinuric stress. In contrast, distal/collecting-duct estimates were not consistent across datasets: GSE241302 was positive, OEP004186 showed a nonsignificant positive estimate, and GSE171458 was near zero and slightly negative. The principal conclusion should therefore remain restricted to PT and loop/TAL-related compartments.

The PT-VCAM1 analysis helped separate a transcriptional-state effect from a change in subtype abundance. PT-VCAM1 cells are frequently interpreted as an injury-associated or adaptive proximal tubular state. In OEP004186, their relative proportion was not increased in MN; the mean proportion was numerically lower and the group difference was nonsignificant. At the same time, the cholesterol-handling score was higher within both conventional PT and PT-VCAM1 cells. The signal therefore did not arise simply because more PT-VCAM1 cells were captured in MN kidneys. It is better interpreted as a program superimposed on more than one proximal tubular state. This distinction is important because recent single-cell studies of MN have emphasized changes in immune-cell abundance, podocyte injury, and cell-cell communication (Shi et al., 2023; Cai et al., 2024; Xu et al., 2021; Gu et al., 2024), whereas the present analysis points to a metabolic program within resident tubular epithelial compartments.

The tissue-level analysis provides an important boundary for interpretation. In GSE200828, the overall program was higher in MN than in living donors, but it was not higher than in FSGS, MCD, Other, or the pooled non-MN disease group. In several direct disease comparisons, the point estimate was lower in MN. This pattern argues against an MN-specific molecular signature and instead suggests a response shared to varying degrees across proteinuric or otherwise diseased kidneys. Systemic dyslipidemia is a common feature of nephrotic syndrome (Vaziri, 2016; Agrawal et al., 2018), and renal lipid dysmetabolism is observed in multiple forms of chronic kidney disease (Mitrofanova et al., 2023). The tubular state identified here may therefore be shaped by the magnitude and duration of proteinuria, the composition of filtered proteins, local inflammation, medication exposure, and baseline metabolic reserve rather than by the initiating autoantigen alone. The glomerular nature of the GSE200828 tissue preparation also means that this dataset supplies disease context, not independent proof of tubular origin.

The HK-2 experiment adds a focused translational bridge. Protein overload increased the expression of two cholesterol-transport genes, ABCA1 and ABCG1, together with the lipid-remodeling genes SREBF1, FASN, and SCD. The magnitude of induction was modest to moderate, ranging from approximately 1.3- to 1.7-fold, but the direction was coherent across both functional branches of the program. This is consistent with earlier evidence that protein exposure can directly alter proximal tubular transcription and that albumin-associated lipid cargo can modulate the severity and character of the response (Abbate et al., 2006; Zoja et al., 2004; Baines and Brunskill, 2011; Ruggiero et al., 2014; Thomas et al., 2002). The model used fatty-acid-free bovine serum albumin, which reduces confounding from exogenous lipid cargo but does not reproduce the full molecular complexity of urinary proteins in MN. The experiment also measured mRNA rather than transporter activity, intracellular cholesterol, lipid droplets, or efflux to extracellular acceptors. It should therefore be regarded as expression-level concordance with protein overload, not functional validation of LXR signaling or cholesterol export.

The paired renal cancer analysis provides a cross-disease context for the experimentally measured five-gene panel. Its coordinated increase across KIRC, KIRP, and KICH, together with patient-level coupling of the cholesterol-efflux and lipid-synthesis/remodeling arms, suggests that the panel captures a broader renal lipid-remodeling response rather than an MN-specific molecular signature. Importantly, individual-gene effects varied by histology, and the TCGA analysis compared tumor with adjacent normal tissue; it therefore cannot be used to infer tumor-cell-intrinsic LXR activation or to validate a causal role for this program in MN. The value of the analysis is contextual: it demonstrates that the experimentally anchored readout behaves as a coordinated multigene state across distinct renal pathological settings. From an oncology perspective, the reproducible paired tumor–normal shift supports the five-gene panel as a tumor-associated molecular readout of renal lipid remodeling and provides a rationale for testing this state in tumor microenvironment and treatment-response studies. The present data, however, do not establish somatic-mutation dependence, immune modulation, immunotherapy response, or resistance mechanisms.

A major strength of the study is the use of the donor as the biological replicate wherever formal inference was made. GSE241302 was treated as exploratory because it contained only one internal normal-reference donor, whereas OEP004186 provided the principal donor-aware comparison. GSE171458 was used for directional support rather than overstated as a definitive validation cohort because it contained only two controls. This hierarchy avoids the common single-cell error of treating thousands of cells from a small number of individuals as thousands of independent samples. Benchmarking studies have shown that differential-expression methods that fail to account for between-replicate variability can generate large numbers of false discoveries (Squair et al., 2021). The present analysis also incorporated donor-specific quality control, pseudobulk aggregation, leave-one-donor-out testing, and leave-one-gene-out analysis, which together strengthen confidence that the principal signal was not produced by one donor or one highly weighted gene.

Several limitations remain. First, the discovery dataset contained only one normal-reference donor, and donor effects cannot be separated from disease effects within GSE241302 alone. Second, although OEP004186 provided a larger, identity-resolved cohort, the analysis remains retrospective and depends on deposited annotations, sample processing, and transcript capture. Third, GSE171458 had only two control donors, resulting in wide confidence intervals. Fourth, the datasets differed in sampling, dissociation, sequencing depth, and annotation, and cross-dataset agreement was stronger at the program level than for every individual gene. Fifth, clinical data on proteinuria burden, treatment status, PLA2R antibody level, serum lipids, and longitudinal kidney function were not sufficiently harmonized to test dose-response or prognostic relationships. Sixth, inferred NR1H2/NR1H3 activity did not survive correction across the full transcription-factor testing space. Finally, the HK-2 experiment involved three replicate measurements and assessed only five transcripts. Confirmation in primary human tubular epithelial cells, organoids, or *ex vivo* tissue, together with protein and lipid measurements, will be necessary to establish the biological consequences of the state. The TCGA component was limited to paired transcriptomic tumor–normal comparisons and did not evaluate somatic mutations, spatial tumor-immune organization, immune-checkpoint treatment exposure, or clinical response and resistance endpoints.

These limitations also define the next steps. Direct measurement of ABCA1 and ABCG1 protein localization, LXR/RXR nuclear signaling, intracellular free and esterified cholesterol, lipid-droplet burden, and cholesterol-efflux capacity would distinguish compensatory transport from lipid accumulation. Experiments comparing fatty-acid-free albumin, lipid-bearing albumin, immunoglobulin-rich protein preparations, and patient-derived urinary proteins could clarify which components of the proteinuric milieu induce the program. Parallel analysis of urine biomarkers and tubular injury markers may help determine whether the state tracks with ongoing protein exposure or tubular stress. Importantly, because the bulk signal was not specific to MN, future studies should include FSGS, MCD, and other proteinuric controls rather than relying only on healthy tissue. For oncology-focused validation, future studies should integrate somatic mutation profiles, single-cell or spatial tumor-immune states, and immunotherapy-treated renal cancer cohorts to determine whether this five-gene lipid-remodeling signature has predictive, resistance-associated, or patient-stratification value.

## Conclusion

In conclusion, donor-aware analysis across multiple kidney single-cell resources identified an LXR-linked cholesterol-handling state in PT and loop/TAL-related epithelial compartments in MN. The state was supported by a multigene architecture, was not explained by PT-VCAM1 expansion, and was reproduced at the expression level in protein-overloaded HK-2 cells. Its presence in other glomerular diseases and the absence of direct lipid-flux measurements indicate that it is best viewed as an MN-associated tubular lipid-homeostasis response within the broader setting of proteinuric kidney injury, rather than as a disease-specific or causally established LXR mechanism. Paired TCGA analysis additionally demonstrated a coordinated five-gene lipid-remodeling increase across KIRC, KIRP, and KICH, extending the relevance of this expression pattern into a renal tumor context. These tumor findings are hypothesis-generating and do not establish mutation dependence, tumor-immune regulation, or therapeutic predictive value.

## Data Availability

GSE241302, GSE171458, and GSE200828 are available through the Gene Expression Omnibus. OEP004186 is available through the National Genomics Data Center/OMIX portal. The frozen program definitions, donor-level score tables, analysis scripts, and figure-source data are available from the corresponding author upon reasonable request. The paired TCGA-KIRC, TCGA-KIRP, and TCGA-KICH expression matrices used for the renal cancer contextual analysis are available through the UCSC Xena GDC hub.
